# The association between endometriosis and risk of endometrial cancer and breast cancer: a meta-analysis

**DOI:** 10.1186/s12905-022-02028-x

**Published:** 2022-11-18

**Authors:** Jiatian Ye, Hongling Peng, Xia Huang, Xiaorong Qi

**Affiliations:** 1 Department of Gynecology and Obstetrics, Key Laboratory of Birth Defects and Related Diseases of Women and Children (Sichuan University), Ministry of Education, West China Second Hospital, Sichuan University, Chengdu, People’s Republic of China; 2 Department of Gynecology and Obstetrics, The Fourth People’s Hospital, Zigong, People’s Republic of China

**Keywords:** Endometriosis, Endometrial cancer, Breast cancer, Risk, Meta-analysis

## Abstract

**Purpose:**

Endometriosis (EMS) is confirmed pathophysiologically to be an estrogen-dependent disease, similar to endometrial hyperplasia/cancer and breast cancer. Epidemiological and biological data on endometriosis might explain links between endometriosis and these cancers. We sought to identify the differences in the risk of endometrial cancer and breast cancer between women with and women without endometriosis.

**Methods:**

We searched PubMed, EMBASE, the Cochrane Library, and four Chinese databases (CNKI, VIP, WanFang, CBM) to identify relevant studies published online between January 2011 and March 2021. In our meta-analysis, we used the Newcastle–Ottawa Scale (NOS) to evaluate the design and quality of all studies, and we calculated the pooled risk ratio (RR) using the random model. The Q test and I^2^ were used to evaluate the degree of heterogeneity of eligible studies. We used funnel plots and Begg’s and Egger’s tests to assess publication bias.

**Results:**

Of the 1369 articles, we finally included 14 cohort studies and seven case–control studies. Data from large cohort and case–control studies indicate that women with endometriosis had an increased risk of both endometrial cancer [RR, 1.662; 95% CI, (1.148–2.407)] and breast cancer [RR, 1.082; 95% CI, (1.001–1.169)].

**Conclusion:**

Endometriosis can increase the risk of endometrial cancer and breast cancer, and women with endometriosis are recommended to receive routine screening in long-term management.

**Supplementary Information:**

The online version contains supplementary material available at 10.1186/s12905-022-02028-x.

## Introduction

Endometriosis (EMS) is a common inflammatory condition defined as endometrial-like tissues found outside of the uterus, mainly in the pelvic area (such as the ovaries, ligaments and peritoneum). Three well-recognized subtypes are named superficial endometriosis (SUP), ovarian endometrioma (OMA), and deep infiltrating endometriosis (DIE) [1]. Pelvic pain and infertility are the two main symptoms that severely impact women’s lives [2]. Because the current diagnosis of EMS requires surgical visualization and the confirmation of pathological results [3], the measurement of the incidence and prevalence of endometriosis is complicated, and estimates vary widely among different studies. Based on the prevalence of pelvic pain and infertility in the general population, the estimated population prevalence of endometriosis is approximately 10% [4, 5] and is higher in symptomatic women [6].

Although endometriosis is a benign gynecological disease, the pattern of its growth is similar to that of malignant disease [7]. The ectopic endometrium, similar to the normal endometrium, has the same reaction to hormones. Abnormal endometrium can adhere and implant into the peritoneum and then proliferate abundantly, which can also lead to invasion of surrounding tissues, such as the bladder and rectum. In addition, the abnormal endometrium shows great power to protect itself from destruction by the immune system [8]. Since first reported by Sampson in 1925 that EMS was associated with malignant tumors, an increasing number of studies have tried to find an association between EMS and cancer. EMS and several malignant tumors have some common risk factors, such as menstrual and reproductive history, cigarette smoking, diet, and environmental exposures [5]; beyond that, some of the treatments for endometriosis, such as physicotherapeutics and medication, also increase the risk of several cancer types [9–11], and Bhyan showed evidence that endometriosis may have shared genetic mechanisms with women’s cancers detected by integrated bioinformatic analysis [12]. Endometriosis is histologically typical and atypical; atypical endometriosis is regarded as the premalignant precursor and has the potential for direct malignant transformation [13–15]. Endometriosis leads to systematic changes, including chronic inflammation, an aberrant immune response or an aberrant milieu, which increases the risk of distal cancer [16].

The retrograde menstruation hypothesis, which is commonly accepted, posits that the mechanism of endometriosis is that eutopic endometrial tissues with molecular defects migrate retrogradely to the abdominal cavity mixed with blood, stick to the peritoneum, and proliferate aggressively, and that endometriosis (like endometrial cancer) can also be regulated by hormones. Therefore, the association between endometriosis and endometrial cancer seems to be noticed by researchers more easily, while breast cancer, which is the other common cancer among reproductive women, can also be influenced by hormone fluctuations. Endometriosis itself and before or after therapy may influence breast cancer directly or indirectly. Epidemiologically, several studies have clearly shown that endometriosis is a risk factor for ovarian cancer [13, 17, 18], but the impact on endometrial cancer and breast cancer is still controversial and even totally converse [18–25]. Several meta-analyses have been published in recent years on the association between endometriosis and cancer; based on 38 cohort studies or case–control studies published before October 24, 2019, Marina Kvaskoff estimated the summary relative risk to be SRR, 1.23; 95% CI, (0.97–1.57), which is not statistically significant, and SRR, 1.04; 95% CI, (1.00–1.09) for the relationship of endometriosis to endometrial cancer and breast cancer, respectively [16]. While this result is different from those of the prior meta-analyses, S. Gandini’s study, based on 32 studies published between 1989 and 2018, suggested that endometriosis confers an increased risk of endometrial cancer [SRR, 1.38, 95% CI (1.10–1.74)], while no association emerged for breast cancer [SRR, 1.04, 95% CI (0.99–1.09)] [26]. Whether endometriosis impacts the risk of these two cancers and the specific physiological and pathological mechanisms involved still need further investigation. Endometriosis, endometrial cancer, and breast cancer are all estrogen-related diseases. Our meta-analysis tried to find their association, and based on the current research fundamentals, we hypothesize that endometriosis can increase the risk of endometrial cancer and breast cancer. Currently, we have limited knowledge of endometriosis, and knowing its association with several cancer types can enhance our understanding of endometriosis pathophysiology, which may advance the treatment and clinical management of endometriosis. Therefore, we performed this meta-analysis to disentangle these intriguing and controversial issues.

## Method

### Search strategy

The reporting of this meta-analysis strictly followed the MOOSE checklist. We comprehensively searched for published relevant observational studies from the databases of PubMed, EMBASE, Cochrane Library, CNKI, VIP, WanFang, and CBM for the past 10 years (from 2011 to March 11, 2021). The search terms were the keywords combined with their corresponding MeSH terms (which are detailed in the supplementary files named “search strategy”). We also searched the references from selected publications to retrieve additional studies that were not identified through electronic searches.

### Selection criteria and exclusion criteria

We included relevant studies that met the following criteria: (1) studies that examined endometriosis (which was diagnosed through self-reports, laparoscopy, surgery or other medical records) and endometrial cancer or breast cancer; (2) human studies and cohort or case–control studies; and (3) publications in which usable risk estimates, such as odds ratios (ORs), risk ratios (RRs), hazard ratios (HRs), and standard incidence ratios (SIRs) with 95% confidence intervals (CIs), were presented or necessary data were given for calculation. (4) If several studies were conducted in the same population, we would select the report with the most applicable estimates or the most recent report. However, we also excluded the following types of studies: (1) meta-analyses, reviews, case reports, editorials, and letters to the editor; (2) animal or cell experiments; (3) studies not published in English or Chinese; and (4) studies not meeting the inclusion criteria.

### Data extraction

Based on the predetermined selection and exclusion criteria, two authors independently extracted data from each study using predesigned forms, and discrepancies were resolved by the third author. For each study, we independently extracted the first author’s name, year of publication, country, age at baseline of the study population, study design, follow-up time of the cohort studies, the sample size of the study, ascertainment of endometriosis and cancer cases, age at the diagnosis of endometriosis, adjustment factors and relative risk estimates with 95% CIs (we chose the model adjusted for the largest number of confounders when different crude and adjusted estimates were reported), and the method of information collection.

### Quality assessment and risk of bias

The Newcastle–Ottawa Scale (NOS) was used to assess the quality of the included cohort and case–control studies [27]. The NOS is composed of three parameters of quality: the selection (four scores at most), comparability (two scores at most), and exposure for a case–control study or outcome for a cohort study (three scores at most). Currently, despite no standard criteria, a study with an NOS score ≥ 7 is considered a high-quality study [13]. Two authors independently evaluated the quality, and discrepancies were resolved by the third author.

### Statistical analysis

We included in this meta-analysis studies reporting different measures of relative risk (RR): single-arm cohort studies (standardized incidence ratio), two-arm cohort studies (rate ratio), and case–control studies (odds ratio). Because the absolute risk of endometrial cancer and breast cancer is low, the three combined measurement methods yield similar relative risk (RR) estimates, and we combined all the RR estimates to ensure the comprehensiveness of the analysis and to enlarge the statistical effectiveness [13]. For the cohort study, when studies had two or more controls, we chose the general population cohort, and when studies had two or more experimental groups, we included the cohort with more populations during the data analysis. The *Q* test and *I*^*2*^ were used to evaluate the degree of heterogeneity of eligible studies. For the *Q* test, *p* > 0.10 was considered representative without statistical heterogeneity, data were interpreted using the fixed effect model, *p* < 0.10 indicated statistically significant heterogeneity, and the random effects model was chosen. For *I*^*2*^, the values of 0, 25, 50, and 75% correspond to no, low, moderate, and high heterogeneity, respectively [28]. We also conducted a subgroup analysis based on the information collection, study design, assessment of endometriosis and cancer, NOS score, and adjustment of confounding factors to evaluate potential sources of heterogeneity. Sensitivity analysis was conducted to evaluate the robustness of the results. We used funnel plots and Begg’s and Egger’s tests to assess publication bias. All statistical analyses were conducted by STATA software, version 16.0.

## Results

### Selection of articles

**Fig. 1:**
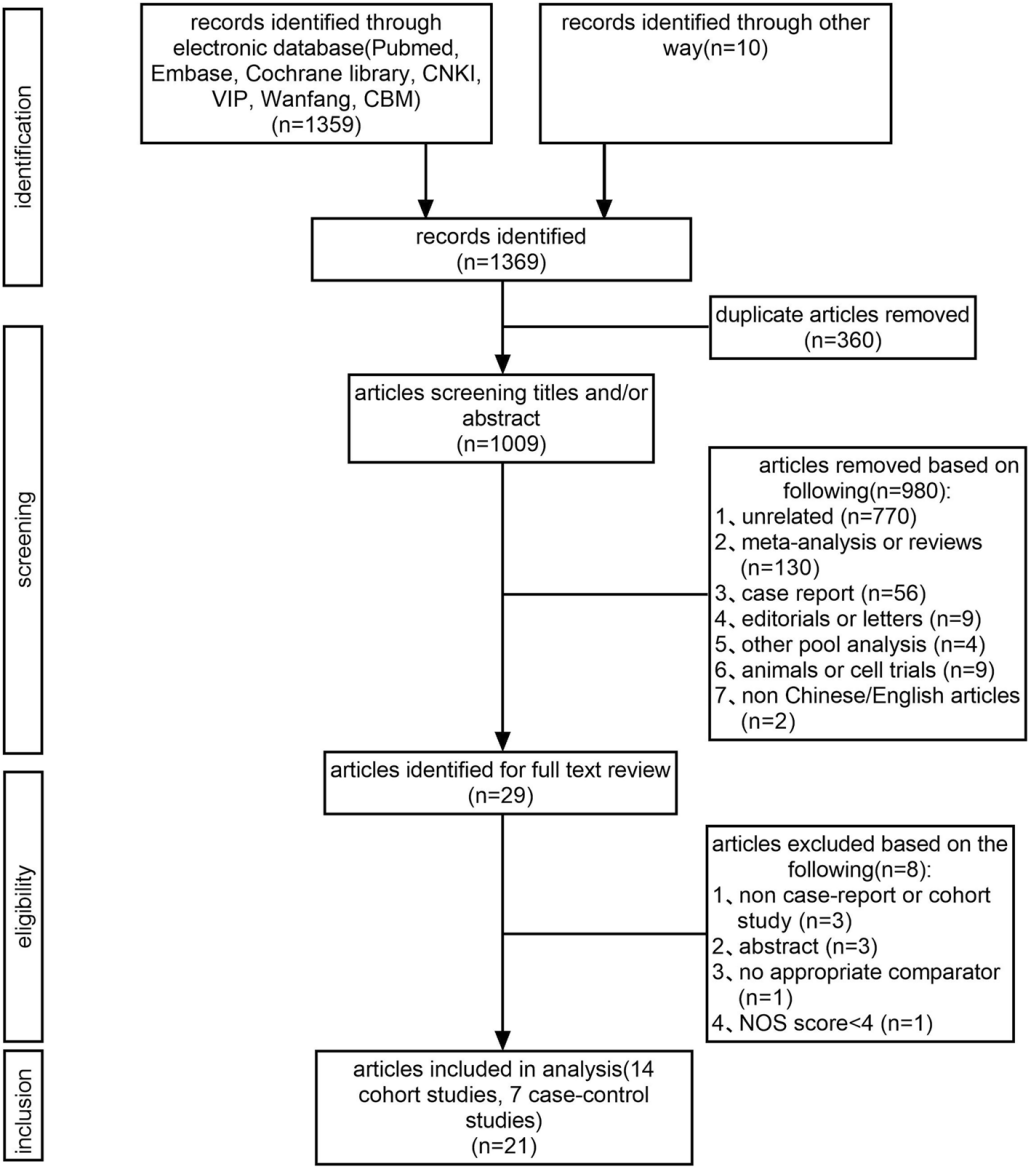
Flow chart for the selection of eligible studies

Two authors independently evaluated the eligibility of studies from the database according to the selection and exclusion criteria, and the third author resolved the disagreement between the two authors after discussion. As a result, a total of 1369 studies were identified. Subsequently, 360 duplicates and 980 unrelated articles were excluded after reviewing the titles and abstracts. Finally, a total of 29 full texts were further assessed, and 8 publications were excluded because they were non-case report or non-cohort studies (*n* = 3), consisted of only an abstract (*n* = 3), had no appropriate comparator (*n* = 1), or had a NOS score of < 4 (n = 1). As a result, 21 studies were included in this meta-analysis, including 14 cohort studies [19–22, 25, 29–37] and 7 case–control studies [23, 24, 38–42]. The flow chart of the study selection is presented in Fig. 1.

### Characteristics of the included studies and quality assessment

A total of 21 articles were included in this analysis, and their characteristics are shown in Table 1. All articles were published between 2011 and 2021. Studies were conducted in America [21, 23, 30, 35, 36, 38], the UK [22, 25], China [29, 33, 34, 37, 39, 41], Korea [19], Denmark [20], Finland [31], Sweden [32], Germany [42], Puerto Rico [24], and Australia [40], with each of the latter seven countries having one study. Regarding the assessment of endometriosis, apart from studies including a clinical diagnosis made by the medical doctors during hospitalization or in the outpatient setting [19, 20], the ICD code of the disease [21, 29, 32–34, 37, 41], medical records from the hospital database or relevant medical documents [39], laparoscopy or surgery [22, 31, 35], and self-reports [23, 24, 30, 36, 38, 40, 42], the remaining study included both patient-reported and clinically reported information [25]. Other than three studies [20, 25, 31], the remaining 18 studies all adjusted several confounders when reporting RR estimates. Thirteen articles were related to endometrial cancer, and 16 articles involved breast cancer (Table 1).

**Table 1:** Characteristics of cohort and case-control studies of the association between endometriosis and endometrial cancer or breast cancer

Study, publication year and country	Study type	follow up time	Average age of baseline (years)	Sample size of the study	Endometriosis assessment	Age at diagnosis of endometriosis (years)	Cancer ascertainment	Adjusted RR(95%CI)	Adjustments	The way of information collection	NOS score
Kyung Jin Eoh, 2021, Korean [19]	cohort study	NA	endometriosis cohort:40.4 ± 8.4 control:44.0 ± 17.0	263,273	ICD code	≥20	ICD code	endometrial cancer: 4.59 (3.56–5.91) breast cancer: 1.44 (1.31–1.58)	Adjusted for age, insurance type, and comorbidities (diabetes, hypertension, hyperlipidemia, chronic obstructive pulmonary disease, chronic kidney disease, liver cirrhosis, and heart)	database	7
Fanghua Shen, 2020, China [39]	case- control study	–	NA	876	medical records	all ages	medical records	endometrial cancer: 0.36 (0.094–1.381)	Adjusted by age at diagnosis and parity	database	6
Frida E. Lundberg, 2019, Sweden [32]	cohort study	control cohort: 23.4 years^a^ infertile cohort: 25.8 years^a^	NA	2,882,847	ICD code	all ages	ICD code	endometrial cancer: 0.94 (0.76–1.17) breast cancer: 1.02 (0.96–1.07)	EC: Adjusted for age, calendar time, education level, country of birth, parity and age at first birth, salpingectomy and bilateral oophorectomy BC: Adjusted for age, calendar time, education level, country of birth, parity and age at first birth, salpingectomy, hysterectomy and bilateral oophorectomy	database	6
Hsing-Chi Hsu, 2019, China [37]	cohort study	NA	nurses cohort:34.0 ± 7.66 control:34.0 ± 7.74	178,870	ICD code	> 20	ICD code	breast cancer: 0.62 (0.22–1.78)	Adjust for study cohort, age, and comorbidities with significant crude HR (cradiovascular disease and diabetes mellitus)	database	7
Liisu Saavalainen, 2018, Finland [31]	cohort study	16.8 years^a^	endometriosis cohort:36.4 ±?	49,933	surgery	10–60	ICD code	breast cancer: 0.99 (0.94–1.03)	–	database	4
Eric S. Surrey, 2018, American [21]	cohort study	NA	endometriosis cohort:36.5 ± 8.2 control:36.4 ± 8.4	134,805	ICD code	18–49	ICD code	endometrial cancer: 2.4 (1.6–3.8) breast cancer: 1.4 (1.1–1.7)	Adjust for age, state, and insurance type. Models also controlled for 15 Charlson–Deyo comorbidities measured during year before index date	database	8
Carrie L Williams, 2018, UK [25]	cohort study	8.8years^a^	endometriosis cohort:34.5 ± 4.8	225,786	patient reported and clinic reported information	all ages	ICD code	endometrial cancer: 0.75 (0.35–1.43) breast cancer: 0.98 (0.86–1.12)	–	database	4
Chih-Ching Yeh, 2018. China [34]	cohort study	NA	NA	120,582	ICD code	all ages	ICD code	endometrial cancer: 1.89 (1.07–3.35) breast cancer:0.99 (0.80–1.23)	Adjusted for the birth year and geographicregion, occupation, urbanization, monthly income, comorbidity	database	6
Leslie V. Farland, 2016, American [35]	cohort study	24 years	endometriosis cohort:35.6 ± 4.2 control:34.3 ± 4.7	16,325	laparoscopy or laparotom	25–42	medical records	breast cancer: 0.96 (0.88–1.06)	Adjust for age, calendar time, family history of breast cancer, age at menarche, body mass index, body mass index at age 18 years, smoking, biopsy confirmed benign breast disease, alcohol intake, recent health seeking behavior, birth weight, parity +age at first birth, breastfeeding, oral contraceptive	questionaire	7
L Saraswat, 2018, UK [22]	cohort study	29 years	endometriosis cohort:32.1 ± 7.3 general population cohort:32.1 ± 7.3	281,937	laparoscopy or laparotom	all ages	ICD code	endometrial cancer: 1.14 (0.57–2.28) breast cancer: 1.28 (1.06–1.54)	Adjust for age, socio-economic status and duration of follow up	database	6
Elizabeth M. Poole, 2017, America [36]	cohort study	18 years	endometriosis cohort:44.5 ± 4.5^b^ control:44.5 ± 4.6^b^	107,721	self-report	all ages	medical records	endometrial cancer: 0.74 (0.39–1.42)	Adjusted for BMI, parity, duration of post-menopausal hormones (by type), age at menopause, age at menarche, menstrual irregularity, infertility history, and duration of oral contraceptive use	questionaire	7
Julie Brøchner Mogensen, 2016, Denmark [20]	cohort study	642,403 person-years	NA	45,790	clinical diagnoses	25–49	ICD code	endometrial cancer: 2.13 (1.77–2.55) breast cancer: 1.05 (1.00–1.11)	_	database	6
Hann-Chin Yu, 2015, China [33]	cohort study	10 years	_	139,392	ICD code	all ages	ICD code	endometrial cancer: 2.83 (1.49–5.35)	Adjusted HRs were adjusted for patients’ age, urbanization level, monthly income, geographic region, hypertension, hyperlipidemia, obesity, and diabetes mellitus	database	8
Victor C. Kok, 2015, China [29]	cohort study	endometriosis cohort:9842 patients-years control cohort:36274 person-years	_	11,330	ICD code	> 20	ICD code	endometrial cancer: 4.05 (1.20–13.66) breast cancer: 1.15 (0.61–2.15)	Adjusted for age group, diabetes mellitus, chronic kidney disease, liver cirrhosis, rheumatoid arthritis, and use of medroxyprogesterone acetate, norethindrone acetate, danazol, and GnRH agonist	database	8
Stefanie Burghaus, 2015, German [42]	case- control study	–	endometrial cancer cases:65.6 ± 10.5 controls:60.9 ± 9.3	1305	self-report	all ages	histopathology reports	endometrial cancer: 2.63 (1.28–5.41)	Adjusted for age, BMI, oral contraceptive use, pregnancies	questionnaire	6
Shu-Chun Chuang, 2015, China [41]	case- control study	–	–	24,420	ICD code	all ages	ICD code	breast cancer: 1.44 (1.15–1.80)	Adjusted for occupation, screen tests (never, once, and twice or above), and average ambulatory visit per year	database	6
Louise A. Brinton, 2014, American [30]	cohort study	30.0 years^a^	_	12,193	self-report	all ages	self-report and medical records	breast cancer: 1.12 (0.93–1.35)	Adjusted for study site, calendar year of first infertility evaluation and gravidity at first clinic visit	database and questionnaire	4
Jaime L. Matta, 2013, American [23]	case- control study	–	breast cancer cases:56.3 ±? controls:52.1 ±?	991	self-report	all ages	histopathology reports	breast cancer: 0.5 (0.30–0.90)	Adjusted by age, BMI, family history of breast cancer, menopause, alcohol use, smoking, multivitamin use, marital status, and saturated fat consumption	questionnaire	7
Luisa Morales, 2013, Puerto Rico [24]	case- control study	–	breast cancer cases:56.4 ± 12.6 controls:52.3 ± 12.5	1126	self-report	all ages	histopathology reports	breast cancer: 0.61 (0.30–1.00)	Adjusted by age, BMI, family history of breast cancer, menopause, number of children, alcohol use, smoking, vitamin use	questionnaire	4
Ingrid J. Rowlands, 2011, Australia [40]	case- control study	–	endometrial cancer cases:61.29 ± 9.5 controls:60.83 ± 9.8	2938	self-report	all ages	histopathology reports	endometrial cancer: 1.47 (1.00–2.17)	Adjusted for age (in years), age at menarche (in years), parous (no, yes), duration of OC use (never, b 60 months; ≥60 months), HRT use (≥ 3 months,< 3 months), smoking (ever, never), BMI (kg/m2)	questionnaire	6
Hazel B. Nichols, 2011, American [38]	case- control study	–	breast cancer cases:66.2 ± 7.4 controls:65.0 ± 7.5	10,046	self-report	all ages	medical records	breast cancer: 0.99 (0.80–1.21)	Adjusted for age, US state, age at menarche, duration of oral contraceptive use, parity, age at first birth, age at menopause, postmenopausal hormone use, body mass index, mammography screening, and family history of breast cancer	telephone interview	7

*NA* Information not available, *RR* Relative risk, *CI* Confidence interval;-^a^ medium follow-up time;-^b^ at midpoint of follow-up

The NOS scores included in this analysis ranged from 4 to 8 after we excluded the studies in which NOS scores were < 4. For cohort studies, 7 articles were of high quality, with an average score of 6.3. For case–control studies, 2 articles were of high quality, with an average score of 6.

### Outcomes

#### Endometrial cancer

**Fig. 2:**
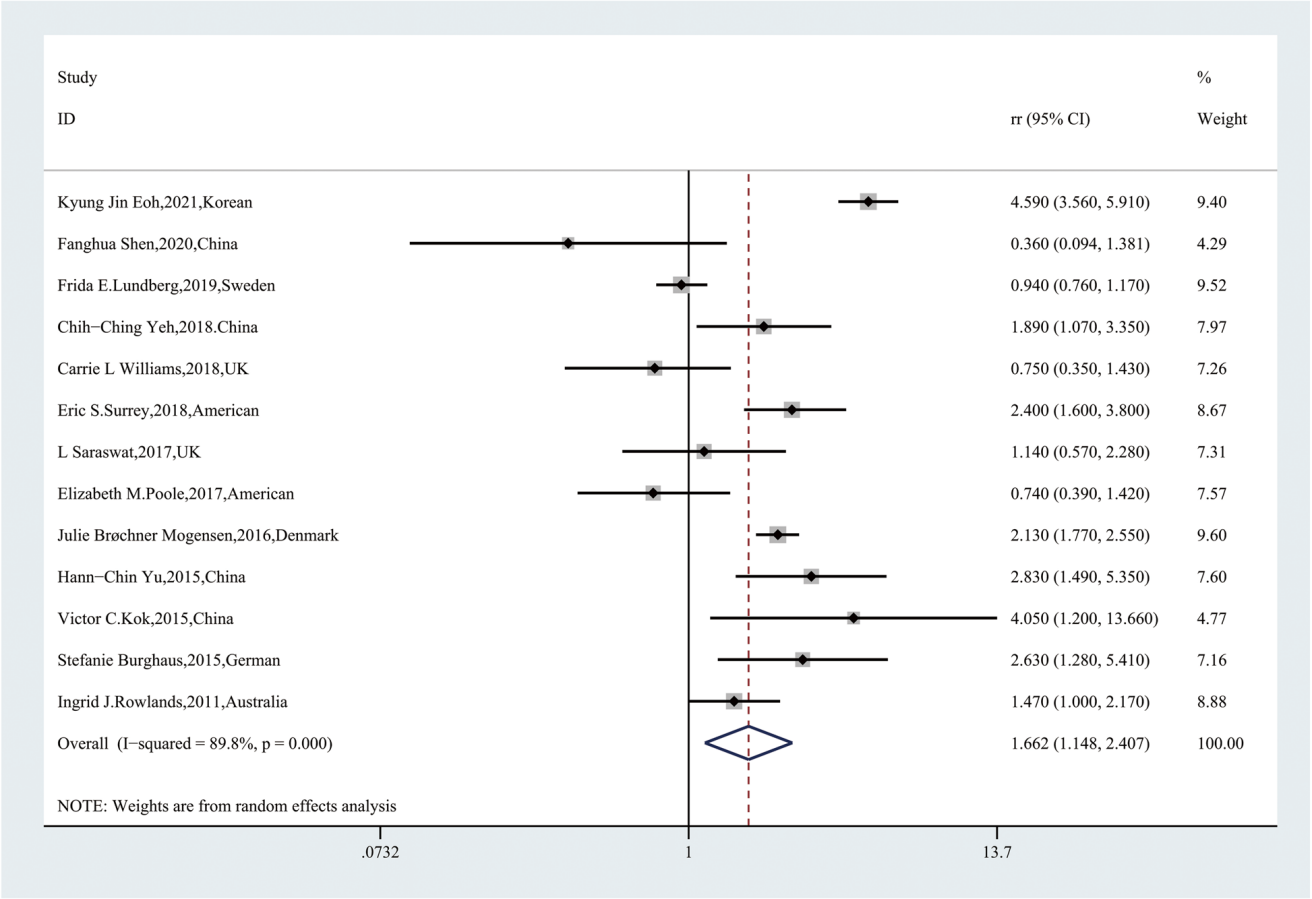
Forest plot of the 13 included studies evaluating the association between endometriosis and endometrial cancer

Thirteen articles were included in evaluating the risk relationship between endometriosis and endometrial cancer. In these 13 studies, endometriosis was associated with a significantly increased risk of endometrial cancer [RR, 1.662; 95% CI, (1.148–2.407)] (Fig. 2), while we found high heterogeneity (*Q* = 118.10, *P* = 0.000; *I*^2^ = 89.8%). We also performed a subgroup analysis to identify the cause of heterogeneity. In the group stratified by the ascertainment of endometrial cancer, ascertainment based on histopathology reports (*P* = 0.163; *I*^2^ = 48.5) and other methods (*P* = 0.344; *I*^2^ = 0.0) showed low heterogeneity (Table 2), which suggested that the method of identification of endometrial cancer may be one of the sources of heterogeneity.

**Table 2:** Summary relative risks and 95%CI for the association between endometriosis and endometrial cancer by study characteristics

Subgroup	No.of studies	Pooled RR(95%CI)		Heterogeneity	
		Random effect	Fixed effect	P	I^2^(%)
**The way of information collection**					
questionnaire	3	1.403 (0.762,2.584)	1.402 (1.036,1.895)	0.034	70.3
database	10	1.743 (1.119,2.716)	1.921 (1.721,2.143)	0.000	91.6
**Study design**					
Case-control studies	3	1.358 (0.622,2.963))	1.526 (1.096,2.124)	0.036	70.0
Cohort studies	10	1.755 (1.139,2.705)	1.890 (1.696,2.107)	0.000	91.8
**Endometriosis assessment**					
Self-report	3	1.403 (0.762,2.584)	1.470 (1.000,2.170)	0.034	70.3
surgery	1	1.140 (0.570,2.280)	1.140 (0.570,2.280)	–	–
ICD	5	2.389 (1.184,4.820)	1.953 (1.692,2.254)	0.000	94.500
Other	4	0.964 (0.347,2.677)	1.937 (1.626,2.308)	0.000	85.800
**Ascertainment of EC**					
Histopathology reports	2	1.810 (1.048,3.127)	1.675 (1.191,2.356)	0.163	48.5
ICD	9	1.922 (1.227,3.012)	1.942 (1.740,2.168)	0.000	92.1
Other	2	0.646 (0.361,1.157)	0.646 (0.361,1.157)	0.344	0.0
**NOS score**					
< 7	8	1.336 (0.918,1.944)	1.486 (1.316,1.678)	0.000	84.0
≥ 7	5	2.457 (1.298,4.652)	3.252 (2.678,3.951)	0.000	86.4
**Adjustment for age**					
Yes	11	1.718 (1.071,2.756)	1.782 (1.570,2.023)	0.000	90.8
No	2	1.339 (0.484,3.701)	1.994 (1.671,2.380)	0.005	87.4
**Adjustment for BMI**					
Yes	3	1.403 (0.762,2.584)	1.402 (1.036,1.895)	0.034	70.3
No	10	1.743(1.119,2.716)	1.921 (1.721,2.143)	0.000	91.6
**Adjustment for oral contraceptive use history**					
Yes	4	1.645 (0.903,2.998)	1.490 (1.112,1.998)	0.023	68.4
No	9	1.644 (1.037,2.604)	1.909 (1.710,2.131)	0.000	92.5
**Adjustment for pregnancies**					
Yes	5	1.116 (0.727,1.713)	1.056 (0.888,1.257)	0.008	70.8
No	8	2.164 (1.470,3.185)	2.506 (2.205,2.847)	0.000	83.7
**Adjustment for smoking**					
Yes	1	1.470 (0.998,2.165)	1.470 (0.998,2.165)	–	–
No	12	1.677 (1.120,2.512)	1.884 (1.693,2.097)	0.000	90.6

**Fig. 3:**
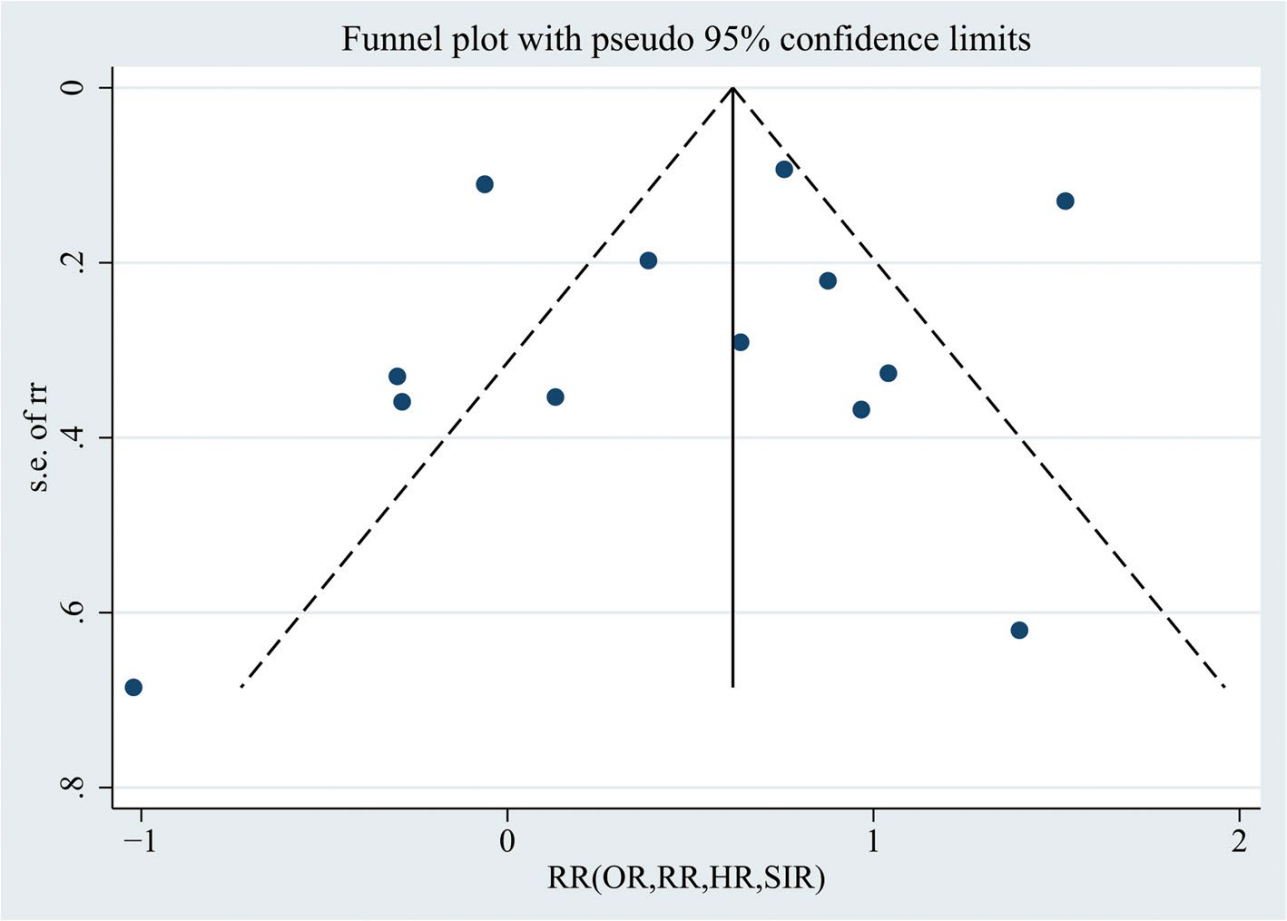
Funnel plot using data from the 13 studies evaluating the association between endometriosis and endometrial cancer

Publication bias was assessed by the funnel plot, as shown in Fig. 3. The funnel plot was visually symmetric. We used Begg’s and Egger’s tests to assess the symmetry of the funnel plot. The *p value*s for Begg’s and Egger’s tests were *p* = 0.502 (> 0.05) and *p* = 0.629 (> 0.05), respectively, suggesting that there was no publication bias of the included studies.

**Fig. 4:**
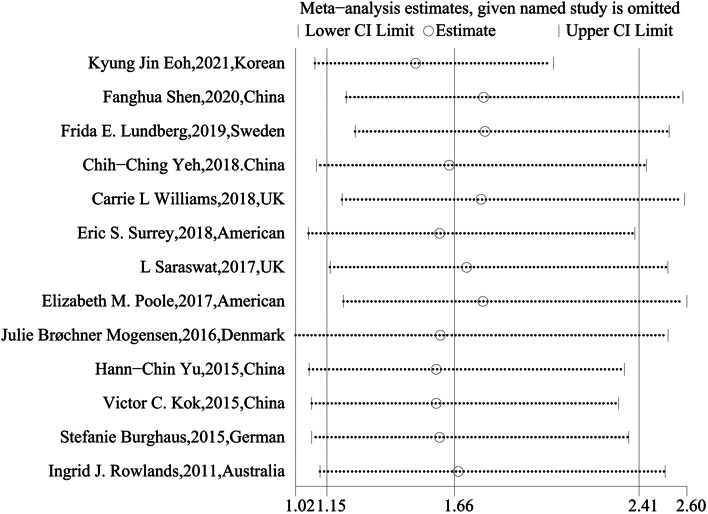
Sensitivity analysis of the 13 studies evaluating the association between endometriosis and endometrial cancer

We used leave-one-out sensitivity analysis to evaluate whether any small study effect influenced the pooled effect size. As a result, no significant changes were observed in the sensitivity analysis (Fig. 4), suggesting that this meta-analysis is stable.

#### Breast cancer

**Fig. 5:**
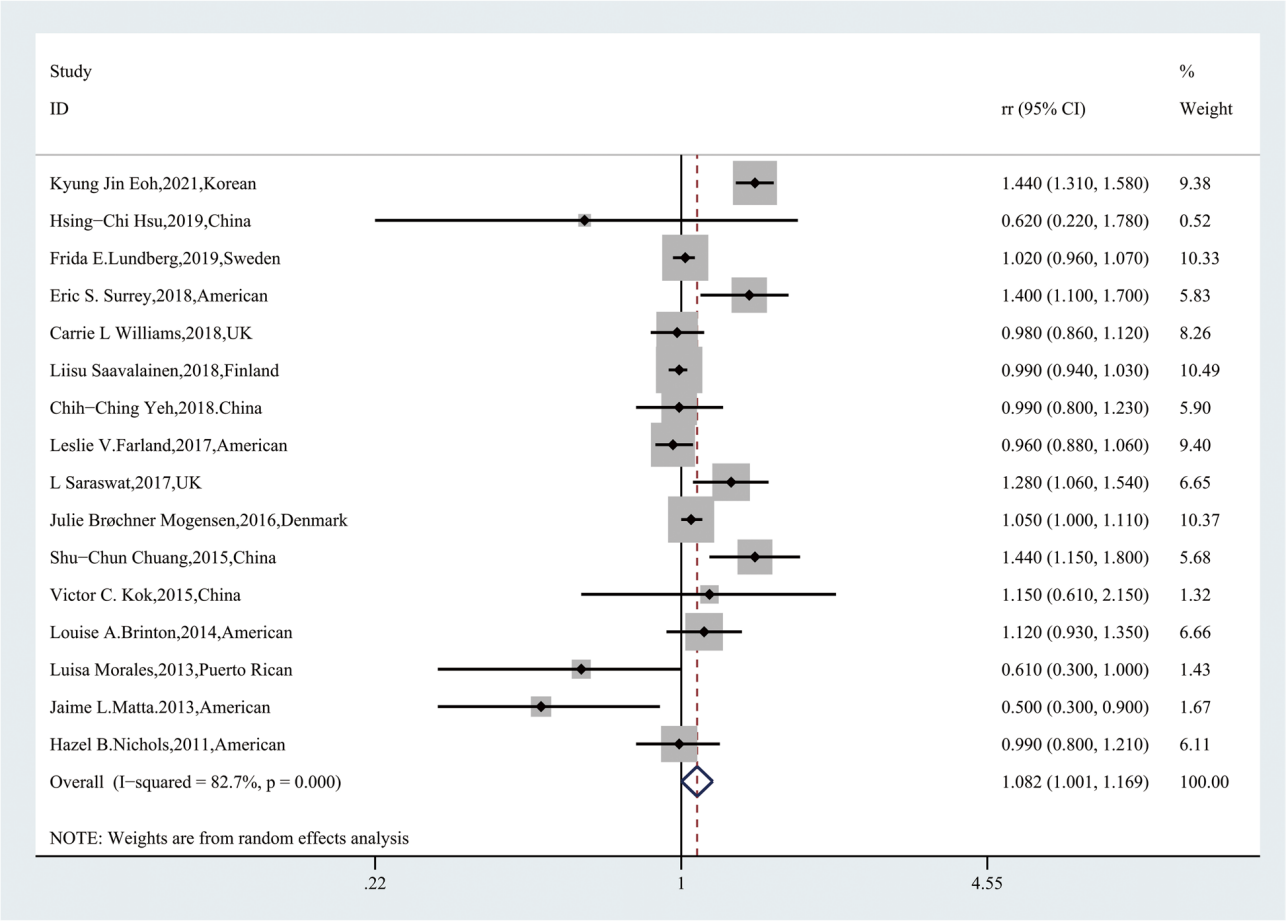
Forest plot of the 16 included studies evaluating the association between endometriosis and breast cancer

A total of 16 articles were included in evaluating the influence of endometriosis on breast cancer. In these 16 studies, endometriosis increased the risk of breast cancer [RR, 1.082; 95% CI, (1.001–1.169)] (Fig. 5), but we also found high heterogeneity within the group (*Q* = 86.62, *P* = 0.000; *I*^2^ = 82.7%). In the subgroup analysis, the group stratified by the ascertainment of breast cancer, ascertainment based on histopathology reports (*P* = 0.632; *I*^2^ = 0) and other methods (*P* = 0.349; *I*^2^ = 5.0) showed low heterogeneity. Studies adjusted for oral contraceptive use and pregnancies also showed low histopathology, which may have been the source of heterogeneity (Table 3).

**Table 3:** Summary relative risks and 95%CI for the association between endometriosis and breast cancer by study characteristics

Subgroup	No.of studies	Pooled RR(95%CI)		Heterogeneity	
		Random effect	Fixed effect	*P*	I^2^(%)
	16	1.082 (1.001,1.169)	1.048 (1.022,1.074)	*0.000*	82.7
**The way of information collection**					
questionnaire	3	0.711 (0.450,1.124)	0.933 (0.852,1.022)	0.027	72.3
database	11	1.134 (1.037,1.241)	1.058 (1.030,1.086)	0.000	86.1
both questionnaire and database	1	1.120 (0.930,1.349)	1.120 (0.930,1.350)	–	–
telephone interview	1	0.990 (0.805,1.218)	0.990 (0.805,1.219)	–	–
**Study design**					
Case-control studies	4	0.877 (1.001,1.169)	1.071 (0.929,1.234)	0.000	83.3
Cohort studies	12	1.094 (1.011,1.183)	1.047 (1.021,1.074)	0.000	83.9
**Endometriosis assessment**					
Self-report	4	0.859 (0.642,1.149)	0.989 (0.868,1.128)	0.018	70.3
surgery	3	1.030 (0.925,1.148)	0.996 (0.957,1.037)	0.022	73.7
ICD	7	1.206 (1.000,1.455)	1.127 (1.079,1.178)	0.000	88.1
Other	2	1.040 (0.991,1.092)	1.040 (0.991,1.092)	0.341	0.0
**Ascertainment of breast cancer**					
Histopathology reports	2	0.547 (0.365,0.821)	0.547 (0.365,0.821)	0.632	0.0
ICD	11	1.134 (1.037,1.241)	1.058 (1.030,1.086)	0.000	86.1
Other	3	0.992 (0.915,1.076)	0.990 (0.916,1.069)	0.349	5.0
**NOS score**					
<7	9	1.051 (0.991,1.114)	1.026 (0.999,1.055)	0.006	63.0
≥ 7	7	1.052 (0.836,1.327)	1.159 (1.091,1.230)	0.000	88.4
**Adjustment for age**					
Yes	11	1.060 (0.924,1.217)	1.083 (1.042,1.125)	0.000	85.4
No	5	1.055 (0.977,1.138)	1.024 (0.991,1.058)	0.010	69.7
**Adjustment for BMI**					
Yes	4	0.860 (0.696,1.164)	0.942 (0.867,1.024)	0.058	59.9
No	12	1.133 (1.041,1.233)	1.059 (1.032,1.087)	0.000	84.8
**Adjustment for oral contraceptive use history**					
Yes	3	0.968 (0.890,1.053)	1.968 (0.890,1.053)	0.834	0.000
No	13	1.101 (1.008,1.203)	1.056 (1.029,1.084)	0.000	85.5
**Adjustment for pregnancies**					
Yes	3	1.004 (0.959,1.051)	1.004 (0.959,1.052)	0.539	0.000
No	13	1.108 (0.998,1.231)	1.067 (1.036,1.099)	0.000	85.1
**Adjustment for smoking**					
Yes	3	0.711 (0.450,1.124)	0.933 (0.852,1.022)	0.027	72.3
No	13	1.122 (1.035,1.216)	1.058 (1.031,1.085)	0.000	83.5
**Adjustment for family history of breast cancer**					
Yes	4	0.860 (0.696,1.064)	0.942 (0.867,1.024)	0.058	59.9
No	12	1.133 (1.041,1.233)	1.059 (1.032,1.087)	0.000	84.8

**Fig. 6:**
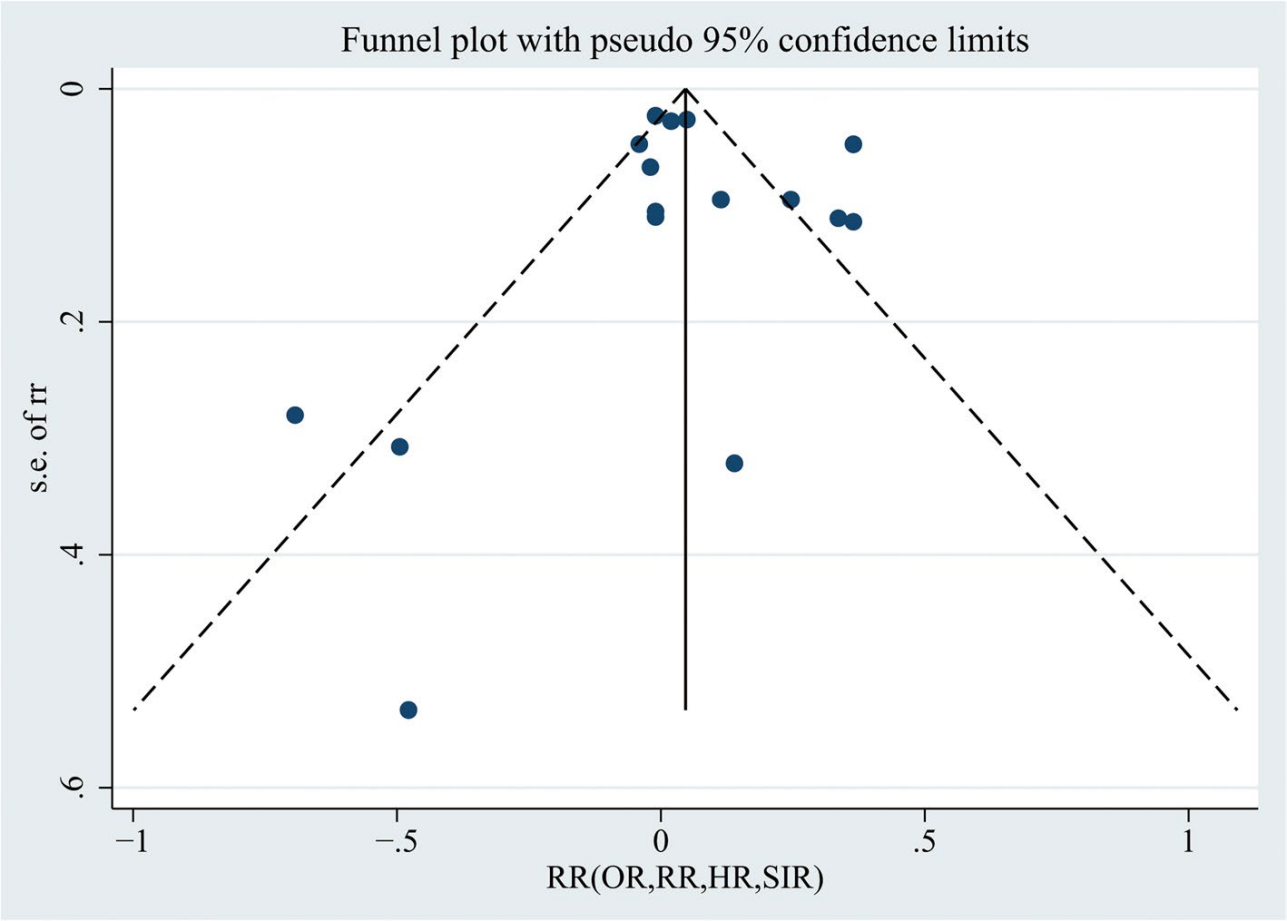
Funnel plot using data from the 16 studies evaluating the association between endometriosis and breast cancer

**Fig. 7:**
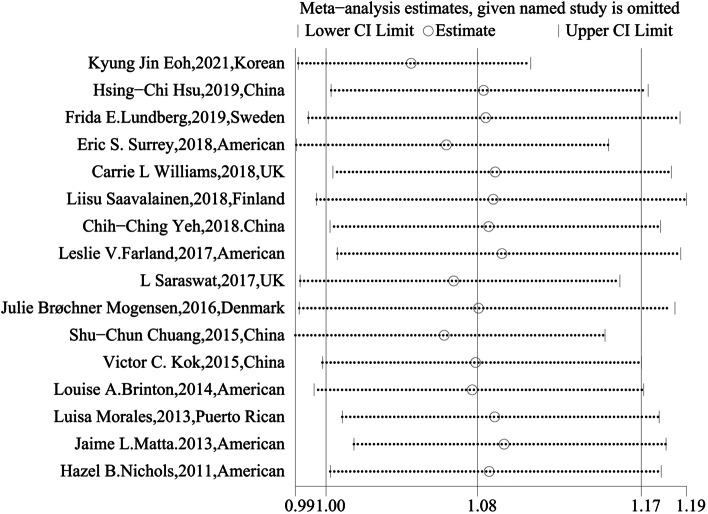
Sensitivity analysis of the 16 studies evaluating the association between endometriosis and breast cancer

In Fig. 6, the funnel plot was visually symmetric, and the *p value*s for Begg’s and Egger’s tests were *p* = 0.499 (> 0.05) and *p* = 0.698 (> 0.05), respectively, suggesting that there was no publication bias. In the leave-one-out sensitivity analysis, no significant changes were observed (Fig. 7), suggesting that this meta-analysis is stable.

## Discussion

Many studies have attempted to find an association between endometriosis and endometrial cancer or breast cancer, but the results are still controversial among different studies [13, 16, 26]. After excluding a low-quality study in which the NOS score was < 4, the NOS scores of the remaining studies ranged from 4 to 8, indicating moderate quality. Our meta-analysis showed that endometriosis can increase the risk of endometrial cancer and that this increase is statistically significant [RR, 1.662; 95% CI, (1.148–2.407)]; a slightly increased risk can also be found in breast cancer [RR, 1.082; 95% CI, (1.001–1.169)]. For patients with endometriosis, besides medication and laparoscopic surgery used to relieve symptoms and remove the lesion, long-term management is crucial [43]. For people with high risks of breast cancer, breast ultrasound (< 40 years) and mammograms (> 40 years) are recommended to screen for breast cancer, while regarding endometrial cancer, screening asymptomatic women for this disease is only recommended for those with Lynch syndrome (LS) [44]. In low- and mid-income countries, because of late diagnosis along with unhealthy lifestyles and eating habits, the burden of breast cancer is increasing exponentially; moreover, “no symptoms” and “no awareness of where to be screened” are among the major reasons for poor screening [45, 46]. The results of our study suggest that women with endometriosis have an increased risk of endometrial cancer and breast cancer; therefore, regular screening may be recommended for these individuals through strengthening their long-term management, which may result in the prevent or early detection of endometrial and breast cancer, but the exact method and timing still need further investigation.

Inevitably, there are some limitations in our meta-analysis. First, the between-study heterogeneity was significant in our analysis; we could not eliminate it through subgroup analysis, and some subgroups were limited to too few of the included studies, which may decrease the credibility of our results. Second, the diagnostic procedures for endometriosis in the included studies were different, which should also be taken into consideration in the conclusion. Third, most of the included studies are retrospective studies, as the risk of recall bias is inevitable compared with that of randomized controlled trials, with the former having a lower level of clinical evidence.

Although endometriosis can increase the risk of endometrial cancer and breast cancer, the risk is relatively low. Undifferentiated screening of all patients with endometriosis may result in a waste of medical resources and increase the financial and psychological pressure on such individuals. Therefore, whether we can narrow down the increased risk in populations with endometriosis to limit the necessary screening to specific subgroups. The American Fertility Society (AFS) classification system for EMS, proposed in 1979 and revised in 1985, is commonly used in the clinic. According to its total scores, EMS is classified into 4 stages: 1–5 denotes stage I (minimal lesions); 6–15 denotes stage II (mild lesions); 16–40 denotes stage III (moderate lesions); and 41–150 denotes stage IV (severe endometriosis) [47, 48]. A study showed that CA125 levels were higher in stage IV than in other stages [49]. Although CA125 is commonly regarded as a specific tumor biomarker of ovarian cancer [50], it is also increased in other cancers, including breast cancer and endometrial cancer [51–53]. Therefore, we wondered whether endometriosis with a higher stage also confers a higher risk of endometrial cancer and breast cancer. Unfortunately, none of the studies we included in this meta-analysis analyzed the risk stratified by the AFS stage of endometriosis. Whether screening can improve outcomes for patients, including decreasing morbidity and mortality, still needs further exploration. Knowing this connection may be beneficial to the management of endometriosis in the future.

Like endometrial cancer and breast cancer, endometriosis is also an estrogen-dependent disease. In endometriotic tissue, aromatase stimulated by PGE2 (Prostaglandin E2), which is essential in the compounding of estrogen, is present at high levels, as well as a lack of 17β-HSD (17β-hydroxysteroid-dehydrogenase) type 2, which can convert estradiol (E2) to the less potent estrone (E1), leading to the accumulation of excess estrogen [54]. In addition, progesterone, which can antagonize estrogen-driven growth in the endometrium, can increase the level of progesterone in ectopic endometrium; however, we found a hyporesponsiveness to progesterone and a low expression of progesterone receptor (PR) in ectopic endometrium, which can also be found in the eutopic endometrium [55]. The excess accumulation of estrogen and the accompanying progesterone resistance causes the excess proliferation of ectopic endometrium. Endometrial cancer can be divided into type 1 and type 2. Type 1 endometrial cancer is endometrioid and estrogen-sensitive, which constitutes 80–85% of all endometrial cancers. Type 2 tumors are estrogen-independent and have a poor prognosis. A study that we included in our analysis showed that endometriosis can increase the risk of type 1 endometrial cancer [SIR, 1.54; 95% CI (1.20–1.96)], while the association cannot be found in type 2 tumors [SIR, 1.06; 95% CI (0.28–2.71) ][20]. Breast cancer is the most frequently diagnosed cancer among women, and one of its risk factors is increasing exposure of breast tissue to estrogen [56]. Based on the hormone receptor status, we can divide breast cancer into three categories: estrogen and progesterone receptor-positive (ER+/PR+) breast cancer, ER+/PR- breast cancer, and ER−/BR- breast cancer (ER−/PR+ has been indicated to not be a reproducible subtype). A study we included showed that endometriosis can increase the risk of ER+/PR- breast cancer [aHR, 1.90; 95% CI (1.44–2.50)] while having no association with the other two cancer types [35]. Five estrogen-responsive genes, CYP19A1, EGFR, ESR2, FOS, and IGF1, were found to be modified in human endometriosis, uterine tumor and breast tumor tissues. We can speculate that estrogen may play an important role between endometriosis and the increasing risk of endometrial cancer and breast cancer, but the study that researched the association between endometriosis and specific types of cancer is not sufficient, and the corresponding result may be contingent. Although we have not found an increasing level of E2 in the serum of populations with endometriosis [57], we have detected a local increase in estrogen in breast cancer and endometrial cancer tissue [58, 59]; as an inflammatory condition, endometriosis may promote the accumulation of E2 in local tissue through the action of a series of inflammatory factors such as IL-1, IL-6, IL-8, PGE, etc., and the exact mechanism is worthy of further exploration.

For endometriosis populations, the combined oral contraceptive pill (COCP) is the first-line drug to relieve symptoms and is widely used in adolescents < 16 years of age. COCP is a compound preparation of a certain amount of estrogen and progesterone that can directly act on the endometrium and can simultaneously act on the hypothalamus through negative feedback and inhibit the secretion of gonadotropin-releasing hormone (GnRH). Studies show that COCP can decrease the risk of endometrial cancer, which means that for people with endometriosis, it may protect the endometrium from malignant transformation [60, 61], but for breast cancer, the result is still controversial. Some meta-analyses have shown that COCP has no association with an increased risk of breast cancer [62], but many studies have shown that endometriosis can increase the risk of breast cancer. The differing results may be related to the age and the duration of oral contraceptives, as well as the years elapsed after stopping such treatment [63–65]. Louise ‘s study showed that for patients < 50 years of age, any OC use before age 20 can increase breast cancer events by approximately threefold, but in patients ≥50 years of age with estrogen receptor-positive tumors, previous OC use at any age can significantly decrease the risk of breast cancer events among patients [66], which may account for the increasing risk between endometriosis and breast cancer for some people. However, restricted to the original research, we cannot perform a further stratification study based on these factors. For individuals who have not accepted any treatment or undergone other medical or surgical therapies, no study shows whether there are any differences in the risks of breast cancer or endometrial cancer. Therefore, knowing the association may be important for the choice of time and methods of endometriosis treatment.

## Conclusion

Knowing the association between endometriosis and cancer has important public and prevalent clinical implications. Our meta-analysis clearly showed that endometriosis can increase the risk of endometrial cancer and breast cancer, which may be significant for long-term management, but we cannot ignore the between-study heterogeneity, which may influence the credibility of the results of our study. For future research, we should perform further stratification research based on the AFS stage or macrophenotype, restricting the increased risk of cancer to specific populations, which may be more valuable for regular screening.

## Supplementary Information

**Additional file 1.** Supplementary files NO.1 Basic characteristics and quality evaluation.

**Additional file 2.** Supplementary files NO.2 Search strategy.

**Additional file 3.** Supplementary files NO.3 Stata analysis.

## Data Availability

All data generated or analyzed during this study are included in this published article (and its supplementary information files).

## Abbreviations

EMS: Endometriosis
SUP: Superficial endometriosis
OMA: Ovarian endometrioma
DIE: Deep infiltrating endometriosis
PGE2: Prostaglandin E2
17β-HSD: 17β-hydroxysteroid-dehydrogenase
PR: Progesterone receptor
COCP: Combined oral contraceptive pill
