# Supplementary material for: The association between endometriosis and risk of endometrial cancer and breast cancer: a meta-analysis
Source: BMC Womens Health. 2022 Nov 18;22:455. doi: 10.1186/s12905-022-02028-x (PMC9673303; doi:10.1186/s12905-022-02028-x)
Supplement: Supplementary file 2 — Additional file 2. Supplementary files NO.2 Search strategy. [file 12905_2022_2028_MOESM2_ESM.docx]

**Supplementary files NO.2 Search strategy**

Embase：

**Endometriosis and endometrial cancer:**

| No. | Query | Results | Date |
| --- | --- | --- | --- |
| #12 | #3 AND #6 AND #10 AND #11 | 834 | 11-Mar-21 |
| #11 | [2011-2021]/py | 15581495 | 11-Mar-21 |
| #10 | 'risk':ab,ti OR 'mortality':ab,ti OR 'cohort':ab,ti OR 'case-control':ab,ti | 4518150 | 11-Mar-21 |
| #6 | #4 OR #5 | 73847 | 11-Mar-21 |
| #5 | 'endometrial neoplasm':ab,ti OR 'neoplasm, endometrial':ab,ti OR 'neoplasms, endometrial':ab,ti OR 'endometrial carcinoma':ab,ti OR 'carcinoma, endometrial':ab,ti OR 'carcinomas, endometrial':ab,ti OR 'endometrial carcinomas':ab,ti OR 'endometrial cancer':ab,ti OR 'cancer, endometrial':ab,ti OR 'cancers, endometrial':ab,ti OR 'endometrial cancers':ab,ti OR 'endometrium cancer':ab,ti OR 'cancer, endometrium':ab,ti OR 'cancers, endometrium':ab,ti OR 'cancer of the endometrium':ab,ti OR 'carcinoma of endometrium':ab,ti OR 'endometrium carcinoma':ab,ti OR 'endometrium carcinomas':ab,ti OR 'cancer of endometrium':ab,ti OR 'endometrium cancers':ab,ti | 38478 | 11-Mar-21 |
| #4 | 'endometrium tumor'/exp | 69146 | 11-Mar-21 |
| #3 | #1 OR #2 | 42267 | 11-Mar-21 |
| #2 | 'endometrioses':ab,ti OR 'endometrioma':ab,ti OR 'endometriomas':ab,ti | 4177 | 11-Mar-21 |
| #1 | 'endometriosis'/exp | 40751 | 11-Mar-21 |

**Endometriosis and breast cancer:**

| No. | Query | Results | Date |
| --- | --- | --- | --- |
| #13 | #3 AND #9 AND #10 AND #11 | 204 | 11-Mar-21 |
| #11 | [2011-2021]/py | 15581495 | 11-Mar-21 |
| #10 | 'risk':ab,ti OR 'mortality':ab,ti OR 'cohort':ab,ti OR 'case-control':ab,ti | 4518150 | 11-Mar-21 |
| #9 | #7 OR #8 | 629313 | 11-Mar-21 |
| #8 | 'breast neoplasm':ab,ti OR 'neoplasm, breast':ab,ti OR 'breast tumors':ab,ti OR 'breast tumor':ab,ti OR 'tumor, breast':ab,ti OR 'tumors, breast':ab,ti OR 'neoplasms, breast':ab,ti OR 'breast cancer':ab,ti OR 'cancer, breast':ab,ti OR 'mammary cancer':ab,ti OR 'cancer, mammary':ab,ti OR 'cancers, mammary':ab,ti OR 'mammary cancers':ab,ti OR 'malignant neoplasm of breast':ab,ti OR 'breast malignant neoplasm':ab,ti OR 'breast malignant neoplasms':ab,ti OR 'malignant tumor of breast':ab,ti OR 'breast malignant tumor':ab,ti OR 'breast malignant tumors':ab,ti OR 'cancer of breast':ab,ti OR 'cancer of the breast':ab,ti OR 'mammary carcinoma, human':ab,ti OR 'carcinoma, human mammary':ab,ti OR 'carcinomas, human mammary':ab,ti OR 'human mammary carcinomas':ab,ti OR 'mammary carcinomas, human':ab,ti OR 'human mammary carcinoma':ab,ti OR 'mammary neoplasms, human':ab,ti OR 'human mammary neoplasm':ab,ti OR 'human mammary neoplasms':ab,ti OR 'neoplasm, human mammary':ab,ti OR 'neoplasms, human mammary':ab,ti OR 'mammary neoplasm, human':ab,ti OR 'breast carcinoma':ab,ti OR 'breast carcinomas':ab,ti OR 'carcinoma, breast':ab,ti OR 'carcinomas, breast':ab,ti | 448511 | 11-Mar-21 |
| #7 | 'breast tumor'/exp | 581909 | 11-Mar-21 |
| #3 | #1 OR #2 | 42267 | 11-Mar-21 |
| #2 | 'endometrioses':ab,ti OR 'endometrioma':ab,ti OR 'endometriomas':ab,ti | 4177 | 11-Mar-21 |
| #1 | 'endometriosis'/exp | 40751 | 11-Mar-21 |

Punmed：

**Endometriosis and endometrial cancer:**

**Search:** **((("Endometriosis"[Mesh]) OR (Endometrioses[Title/Abstract] OR Endometrioma[Title/Abstract] OR Endometriomas[Title/Abstract])) AND (("Endometrial Neoplasms"[Mesh]) OR (((((((((((((((((((Neoplasm, Endometrial[Title/Abstract]) OR (Neoplasms, Endometrial[Title/Abstract])) OR (Endometrial Carcinoma[Title/Abstract])) OR (Carcinoma, Endometrial[Title/Abstract])) OR (Carcinomas, Endometrial[Title/Abstract])) OR (Endometrial Carcinomas[Title/Abstract])) OR (Endometrial Cancer[Title/Abstract])) OR (Cancer, Endometrial[Title/Abstract])) OR (Cancers, Endometrial[Title/Abstract])) OR (Endometrial Cancers[Title/Abstract])) OR (Endometrium Cancer[Title/Abstract])) OR (Cancer, Endometrium[Title/Abstract])) OR (Cancers, Endometrium[Title/Abstract])) OR (Cancer of the Endometrium[Title/Abstract])) OR (Carcinoma of Endometrium[Title/Abstract])) OR (Endometrium Carcinoma[Title/Abstract])) OR (Endometrium Carcinomas[Title/Abstract])) OR (Cancer of Endometrium[Title/Abstract])) OR (Endometrium Cancers[Title/Abstract])))) AND (risk[Title/Abstract] OR risk[MeSH:noexp] OR mortality[Title/Abstract] OR mortality[MeSH:noexp] OR cohort[Title/Abstract])** **OR case-control [Title/Abstract])**Filters: **in the last 10 years**

**Endometriosis and breast cancer:**

**Search:**((("Endometriosis"[Mesh]) OR (Endometrioses[Title/Abstract] OR Endometrioma[Title/Abstract] OR Endometriomas[Title/Abstract])) AND (("Breast Neoplasms"[Mesh]) OR ((((((((((((((((((((((((((((((((((((Breast Neoplasm[Title/Abstract]) OR (Neoplasm, Breast[Title/Abstract])) OR (Breast Tumors[Title/Abstract])) OR (Breast Tumor[Title/Abstract])) OR (Tumor, Breast[Title/Abstract])) OR (Neoplasms, Breast[Title/Abstract])) OR (Breast Cancer[Title/Abstract])) OR (Cancer, Breast[Title/Abstract])) OR (Mammary Cancer[Title/Abstract])) OR (Cancer, Mammary[Title/Abstract])) OR (Cancers, Mammary[Title/Abstract])) OR (Mammary Cancers[Title/Abstract])) OR (Malignant Neoplasm of Breast[Title/Abstract])) OR (Breast Malignant Neoplasm[Title/Abstract])) OR (Breast Malignant Neoplasms[Title/Abstract])) OR (Malignant Tumor of Breast[Title/Abstract])) OR (Breast Malignant Tumor[Title/Abstract])) OR (Breast Malignant Tumors[Title/Abstract])) OR (Cancer of Breast[Title/Abstract])) OR (Cancer of the Breast[Title/Abstract])) OR (Mammary Carcinoma, Human[Title/Abstract])) OR (Carcinoma, Human Mammary[Title/Abstract])) OR (Carcinomas, Human Mammary[Title/Abstract])) OR (Human Mammary Carcinomas[Title/Abstract])) OR (Mammary Carcinomas, Human[Title/Abstract])) OR (Human Mammary Carcinoma[Title/Abstract])) OR (Mammary Neoplasms, Human[Title/Abstract])) OR (Human Mammary Neoplasm[Title/Abstract])) OR (Human Mammary Neoplasms[Title/Abstract])) OR (Neoplasm, Human Mammary[Title/Abstract])) OR (Neoplasms, Human Mammary[Title/Abstract])) OR (Mammary Neoplasm, Human[Title/Abstract])) OR (Breast Carcinoma[Title/Abstract])) OR (Breast Carcinomas[Title/Abstract])) OR (Carcinoma, Breast[Title/Abstract])) OR (Carcinomas, Breast[Title/Abstract])))) AND (risk[Title/Abstract] OR risk[MeSH:noexp] OR mortality[Title/Abstract] OR mortality[MeSH:noexp] OR cohort[Title/Abstract]) OR case-control [Title/Abstract]) Filters: in the last 10 years.

**Cochrane lirrary**

**研究方法：**Register of Controlled Trials (CENTRAL) in the Cochrane Library

| Search Name: | 子宫内膜异位症和子宫内膜癌及乳腺癌相关性 | |
| --- | --- | --- |
| Date Run: | 11/03/2021 15:48:36 | |
| Comment: |  |  |
|  |  |  |
| ID | Search | Hits |
| #1 | MeSH descriptor: [Endometriosis] explode all trees | 867 |
| #2 | (Endometrioses):ti,ab,kw OR (Endometrioma):ti,ab,kw OR (Endometriomas):ti,ab,kw (Word variations have been searched) | 335 |
| #3 | #1 OR #2 | 1093 |
| #4 | MeSH descriptor: [Endometrial Neoplasms] explode all trees | 640 |
| #5 | (Endometrial Neoplasm):ti,ab,kw OR (Neoplasm, Endometrial):ti,ab,kw OR (Neoplasms, Endometrial):ti,ab,kw OR (Endometrial Carcinoma):ti,ab,kw OR (Carcinoma, Endometrial):ti,ab,kw OR (Carcinomas, Endometrial):ti,ab,kw OR (Endometrial Carcinomas):ti,ab,kw OR (Endometrial Cancer):ti,ab,kw OR (Cancer, Endometrial):ti,ab,kw OR (Cancers, Endometrial):ti,ab,kw OR (Endometrial Cancers):ti,ab,kw OR (Endometrium Cancer):ti,ab,kw OR (Cancer, Endometrium):ti,ab,kw OR (Cancers, Endometrium):ti,ab,kw OR (Cancer of the Endometrium):ti,ab,kw OR (Carcinoma of Endometrium):ti,ab,kw OR (Endometrium Carcinoma):ti,ab,kw OR (Endometrium Carcinomas):ti,ab,kw OR (Cancer of Endometrium):ti,ab,kw OR (Endometrium Cancers):ti,ab,kw | 2681 |
| #6 | #4 OR #5 | 2719 |
| #7 | MeSH descriptor: [Breast Neoplasms] explode all trees | 13331 |
| #8 | (Breast Neoplasm):ti,ab,kw OR (Neoplasm, Breast):ti,ab,kw OR (Breast Tumors):ti,ab,kw OR (Breast Tumor):ti,ab,kw OR (Tumor, Breast):ti,ab,kw OR (Tumors, Breast):ti,ab,kw OR (Neoplasms, Breast):ti,ab,kw OR (Breast Cancer):ti,ab,kw OR (Cancer, Breast):ti,ab,kw OR (Mammary Cancer):ti,ab,kw OR (Cancer, Mammary):ti,ab,kw OR (Cancers, Mammary):ti,ab,kw OR (Mammary Cancers):ti,ab,kw OR (Malignant Neoplasm of Breast):ti,ab,kw OR (Breast Malignant Neoplasm):ti,ab,kw OR (Breast Malignant Neoplasms):ti,ab,kw OR (Malignant Tumor of Breast):ti,ab,kw OR (Breast Malignant Tumor):ti,ab,kw OR (Breast Malignant Tumors):ti,ab,kw OR (Cancer of Breast):ti,ab,kw OR (Cancer of the Breast):ti,ab,kw OR (Mammary Carcinoma, Human):ti,ab,kw OR (Carcinoma, Human Mammary):ti,ab,kw OR (Carcinomas, Human Mammary):ti,ab,kw OR (Human Mammary Carcinomas):ti,ab,kw OR (Mammary Carcinomas, Human):ti,ab,kw OR (Human Mammary Carcinoma):ti,ab,kw OR (Mammary Neoplasms, Human):ti,ab,kw OR (Human Mammary Neoplasm):ti,ab,kw OR (Human Mammary Neoplasms):ti,ab,kw OR (Neoplasm, Human Mammary):ti,ab,kw OR (Neoplasms, Human Mammary):ti,ab,kw OR (Mammary Neoplasm, Human):ti,ab,kw OR (Breast Carcinoma):ti,ab,kw OR (Breast Carcinomas):ti,ab,kw OR (Carcinoma, Breast):ti,ab,kw OR (Carcinomas, Breast):ti,ab,kw | 38799 |
| #9 | #7 OR #8 | 38799 |
| #10 | #3 AND #6 | 26 |
| #11 | #3 AND #9 | 4 |


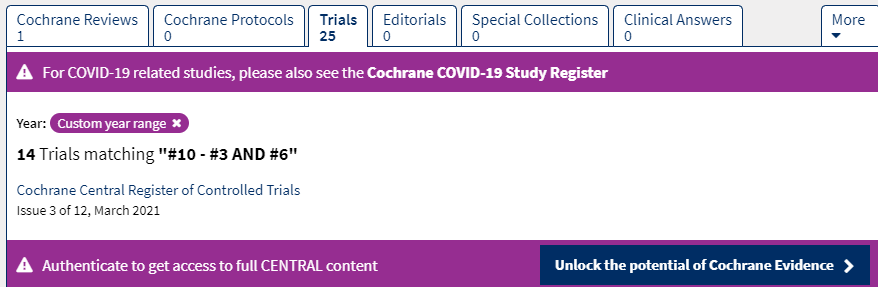


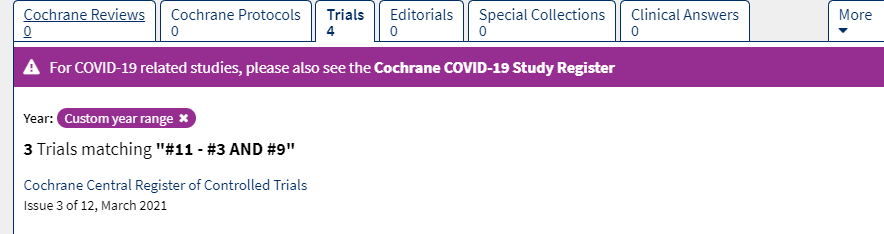


CBM

| 序号 | 检索表达式 | 命中文献数 | 检索时间 |
| --- | --- | --- | --- |
| 1) | "子宫内膜异位症"[不加权:扩展] | 22647 | 54:38.0 |
| 2) | "内异症"[常用字段:智能] OR "子宫内异症"[常用字段:智能] | 2255 | 57:41.0 |
| 3) | (#2) OR (#1) | 22883 | 58:29.0 |
| 4) | "子宫内膜肿瘤"[不加权:扩展] | 14022 | 58:58.0 |
| 5) | "子宫内膜癌"[常用字段:智能] OR "子宫体癌"[常用字段:智能] | 17582 | 59:46.0 |
| 6) | (#5) OR (#4) | 17582 | 59:59.0 |
| 7) | "队列研究"[不加权:扩展] | 624195 | 01:03.0 |
| 8) | "前瞻性研究"[常用字段:智能] | 25031 | 01:42.0 |
| 9) | "病例对照研究"[不加权:扩展] | 409565 | 03:15.0 |
| 10) | "回顾性研究"[常用字段:智能] | 406245 | 03:36.0 |
| 11) | (#10) OR (#9) OR (#8) OR (#7) | 660864 | 03:55.0 |
| 12) | "乳腺肿瘤"[不加权:扩展] | 105060 | 04:40.0 |
| 13) | "乳腺癌"[常用字段:智能] | 118842 | 04:59.0 |
| 14) | (#13) OR (#12) | 118842 | 05:05.0 |
| 15) | ((#11) AND (#6) AND (#3)) AND 2011-2021[日期] | 18 | 05:53.0 |
| 16) | ((#14) AND (#11) AND (#3)) AND 2011-2021[日期] | 0 | 08:15.0 |

VIP：

子宫内膜异位症-子宫内膜癌（0）

检索式：[(((((((((题名或关键词=子宫内膜异位症 OR 题名或关键词=endometriosis) OR 题名或关键词=patients with endometriosis) OR 题名或关键词=pelvic endometriosis) OR 题名或关键词=内异症) OR 题名或关键词=子宫内异症) AND ((((((((题名或关键词=子宫内膜癌 OR 题名或关键词=carcinoma of endometrium) OR 题名或关键词=endometrial cancer) OR 题名或关键词=endometrial carcino ma) OR 题名或关键词=endometrial carcinoma) OR 题名或关键词=endometrial carcinomas) OR 题名或关键词=endometrial neoplasms) OR 题名或关键词=宫体癌) OR 题名或关键词=子宫体癌))) AND ((((((((题名或关键词=回顾性研究 OR 题名或关键词=retrospective stud y) OR 题名或关键词=retrospective study) OR 题名或关键词=病例对照研究) OR 题名或关键词=队列研究) OR 题名或关键词=cohort study) OR 题名或关键词=cohort studies) OR 题名或关键词=cohort study) OR 题名或关键词=前瞻性研究))) AND (years:[2011 TO 2021])](http://qikan.cqvip.com/Qikan/search/index?LngMySearHistoryIdGuid=e5b93f6e-0e96-45c4-bb7d-ac3089eedc63&from=Qikan_Article_History)

子宫内膜异位症-乳腺癌（0）

检索式：[(((((((题名或关键词=子宫内膜异位症 OR 题名或关键词=endometriosis) OR 题名或关键词=patients with endometriosis) OR 题名或关键词=pelvic endometriosis) OR 题名或关键词=内异症) OR 题名或关键词=子宫内异症) AND ((((((题名或关键词=乳腺癌 OR 题名或关键词=breast cancer) OR 题名或关键词=breast carcinoma) OR 题名或关键词=mammary cancer) OR 题名或关键词=乳岩) OR 题名或关键词=乳癌) OR 题名或关键词=乳腺肿瘤)) AND ((((((((任意字段=回顾性研究 OR 任意字段=retrospective stud y) OR 任意字段=retrospective study) OR 任意字段=病例对照研究) OR 任意字段=队列研究) OR 任意字段=cohort study) OR 任意字段=cohort studies) OR 任意字段=cohort study) OR 任意字段=前瞻性研究)) AND (years:[2011 TO 2021])](http://qikan.cqvip.com/Qikan/search/index?LngMySearHistoryIdGuid=c6c08ef2-8cc3-4608-a9ed-89d93c3b0418&from=Qikan_Article_History)

万方：

子宫内膜异位症-子宫内膜癌（11）

 (题名或关键词:(子宫内膜异位症 or 内异症 or 子宫内异症) and 题名或关键词:(子宫内膜癌 or 子宫体癌) and 摘要:(病例对照研究 or 队列研究 or 回顾性研究 or 前瞻性研究)) and Date:2011-2021

子宫内膜异位症-乳腺癌（8）

 (题名或关键词:(子宫内膜异位症 or 内异症 or 子宫内异症) and 题名或关键词:(乳腺癌 or 乳腺肿瘤) and 摘要:(病例对照研究 or 队列研究 or 回顾性研究 or 前瞻性研究)) and Date:2011-2021

知网：

| 检索主题：子宫内膜异位症与子宫内膜癌 |
| --- |
| 检索范围：期刊,外文期刊,报纸,国内会议,国际会议中文,国际会议外文,会议视频,外文会议,博士,硕士,中文图书,国家标准,行业标准,中国标准题录数据库,国外标准题录数据库,职业标准,成果,学术辑刊,古籍,特色期刊 |
| 检索年限：不限 |
| 检索时间：2021-03-11 |
| 检索式A： 主题 = 子宫内膜异位症 AND 主题 = 子宫内膜癌 or ( 题名 = 子宫内膜异位症 or 题名 = 子宫内膜癌 ) or ( title=中英文扩展(子宫内膜异位症) or title=中英文扩展(子宫内膜癌) ) or ( v_subject=中英文扩展(子宫内膜异位症 ) or v_subject=中英文扩展(子宫内膜癌 ) ) AND 摘要=队列研究+病例对照研究+病例对照+对照 or abstract=中英文扩展(队列研究)+中英文扩展(病例对照研究)+中英文扩展(病例对照)+中英文扩展(对照 ) AND 发表时间 Between (2011-01-01,2021-03-11 ) (模糊匹配) |

| 检索主题：子宫内膜异位症与乳腺癌 |
| --- |
| 检索范围：期刊,外文期刊,博士,硕士,国内会议,国际会议中文,国际会议外文,会议视频,外文会议,报纸,国家标准,行业标准,中国标准题录数据库,国外标准题录数据库,职业标准,成果,中文图书,特色期刊,学术辑刊 |
| 检索年限：不限 |
| 检索时间：2021-03-11 |
| 检索式A： 主题 = 子宫内膜异位症 AND 主题 = 乳腺癌 AND 主题 = 队列研究+病例对照研究+对照 or ( 题名 = 子宫内膜异位症 or 题名 = 乳腺癌 or 题名 = 队列研究+病例对照研究+对照 ) or ( title=中英文扩展(子宫内膜异位症) or title=中英文扩展(乳腺癌) or title=中英文扩展(队列研究)+中英文扩展(病例对照研究)+中英文扩展(对照 ) ) or ( v_subject=中英文扩展(子宫内膜异位症 ) or v_subject=中英文扩展(乳腺癌 ) or v_subject=中英文扩展(队列研究)+中英文扩展(病例对照研究)+中英文扩展(对照 ) ) AND 发表时间 Between (2011-01-01,2021-03-11 ) (模糊匹配) |
